# A Histopathological Study of the Spectrum of Skin Lesions in a Tertiary Care Hospital: A Retrospective Study

**DOI:** 10.7759/cureus.47164

**Published:** 2023-10-16

**Authors:** Pratibha Dawande, Rashmi Wankhade, Anita B Sajjanar, Nandkishor J Bankar

**Affiliations:** 1 Pathology, Datta Meghe Medical College, Datta Meghe Institute of Higher Education and Research, Nagpur, IND; 2 Microbiology, Jawaharlal Nehru Medical College, Datta Meghe Institute of Higher Education and Research, Wardha, IND

**Keywords:** non-neoplastic lesion, skin biopsy, squamous cell carcinoma, leprosy, histopathology

## Abstract

Background

The skin is the largest organ of the body with many different functions. All age groups are affected by skin diseases, which are widespread in underdeveloped nations. From a straightforward vesicular non-neoplastic lesion to a catastrophic neoplastic lesion, skin disorders exhibit a wide variety of geographic patterns. To make an accurate diagnosis, identify etiological agents, and assist a dermatologist or clinician in selecting the best course of treatment, a skin biopsy must undergo histopathological analysis. The present study was conducted to investigate the histological diagnosis of skin lesions, establish the distribution by age and sex, identify the most prevalent skin lesions, and further subclassify the most prevalent condition.

Methodology

A retrospective, cross-sectional study was conducted in the Department of Pathology at Datta Meghe Medical College, Wanadongari, Nagpur over the course of a year. Hematoxylin and eosin were used to stain a total of 50 skin biopsy samples, with special stain when necessary, and then examined.

Results

The study involved a total of 50 patients, with 39 (78%) males and 11 (22%) females. With 16 (32%) cases in the 21-30-year age group, the early age group preponderance was recorded. Overall, 16 (32%) cases had microbial diseases, followed by eight (16%) cases with non-infectious vesicobullous diseases and vesicopustular disease, and five (10%) cases with non-infectious erythematous papular and squamous disease. In 12 (24%) cases, leprosy was the most prevalent microbiological disease. In five (10%) cases, pemphigus vulgaris was the most prevalent vesicobullous condition. Psoriasis, which was present in two (4%) cases, was the most common non-infectious erythematous papular and squamous disease. Squamous cell carcinoma, which was seen in seven (14%) cases, was the most prevalent neoplastic lesion.

Conclusions

In skin lesions, males outnumbered females. Patients in the younger age groups were most commonly involved. Leprosy and squamous cell carcinoma were, respectively, the most prevalent non-neoplastic and neoplastic skin lesions in our study.

## Introduction

The skin, which accounts for 16% of the body’s weight, is the largest organ. It produces a protective coat, has characteristics tailored to its purpose, and significantly contributes to maintaining homeostasis through its barrier function [1]. In India, skin problems are among the most prevalent health problems, with prevalence ranging from 6.3% to 11.6% [2].

Skin illnesses manifest in a variety of ways, ranging from basic vesicular non-neoplastic lesions to lethal malignant lesions. Various skin conditions range from non-infectious, non-specific, and infectious disorders to different kinds of neoplastic conditions including benign as well as malignant tumors. Dermatological lesions, which cover a broad spectrum, vary from one nation to the next and in different regions of the same nation. This diversity is influenced by a variety of factors, including sex, age, and any accompanying systemic disorders, as well as socioeconomic factors, literacy levels, racial characteristics, and social standards. It affects 6.3% to 11.16% of the population [3].

Due to advances in medical facilities and increased public awareness of skin diseases, histological analysis of clinically diagnosed skin lesions now plays a significant role in the confirmation of the diagnosis [4]. The majority of skin disorders can be identified without using histology by examining the patient’s history, clinical symptoms, and biochemical tests. However, histological examination continues to be the gold standard for helping the dermatologist in resolving diagnostic conundrums [5]. To determine the etiological agents, provide an accurate diagnosis, and assist a dermatologist or clinician in choosing the best course of treatment, a histopathological examination of skin biopsy is required [6]. The present study aims to determine the frequency of different skin lesions in our tertiary care hospital, the role of histopathology in the diagnosis of various neoplastic as well as non-neoplastic skin lesions, and to classify them under various histopathological categories, which aids in better patient management.

## Materials and methods

Study design

This retrospective, cross-sectional study was conducted in the histopathology section of the Department of Pathology at the Datta Meghe Medical College, Wanadongari, Nagpur, for a period of one year from January 2022 to December 2022.

Sample size

A total of 50 skin biopsy samples collected from the Dermatology Outpatient Department and sent to the Department of Pathology for clinical diagnosis were included in the study.

Inclusion and exclusion criteria

The study included all cases that arrived throughout the study period including skin biopsies taken from new cases, previously treated, partially treated, on treatment, and cases of recurrence. Skin biopsies that were insufficient or autolyzed were excluded from the study.

Data collection

Detailed clinical history and data from the requisition form provided by the dermatologist including the name of the patient, age of the patient, gender, clinical presentation, and differential diagnosis were documented.

Study procedure

The biopsy samples from skin lesions that had been clinically diagnosed were sent to the histopathology laboratory in 10% formalin. The sample was fixed in 10% neutral buffer formalin over a period of 12 to 24 hours. All biopsy tissue samples underwent a gross examination, and all measurements were taken. After 3 to 4 mm thick sections were cut, they were stained with hematoxylin and eosin stain. A microscopic examination was performed and histomorphological features were noted. When necessary, special stains such as Fite-Faraco, Ziehl-Neelsen, and periodic acid-Schiff were applied.

Ethical consideration

Ethical approval was obtained from the Institutional Ethics Committee of Shalinitai Meghe Hospital and Research Centre (approval number: SMHRC/IEC/2022/09-35).

Statistical analysis

All data were collected and the results were arranged in a tabulated form. The number and percentage of incidence in different age groups, between genders, among different types of lesions, and in comparison to other studies were calculated by descriptive statistical analysis.

## Results

In this study, 50 cases of skin lesions that occurred over the course of a year were examined and divided into different categories. Table 1 shows that out of 50 skin biopsies 24 (48%) patients had incisional biopsies performed. Overall, 14 (28%) patients were chosen for punch biopsies, and 12 (24%) patients were chosen for excisional biopsies.

**Table 1:** Distribution of cases according to type of biopsy (n = 50).

Type of biopsy	Number of cases	Percentage (%)
Punch	14	28
Incisional	24	48
Excisional	12	24

According to Table 2, most of the cases, 16 (32%), were between the ages of 21 and 30 years, and the next largest group of 10 (20%) patients was between the ages of 31 and 40 years. The age ranged from four years to 80 years. Only nine (18%) patients were under the age of 18 years, while 41 (82%) were adults and elderly. Table 3 shows out of 50 cases, 39 were male and 11 were female, with more male patients than female patients.

**Table 2:** Age distribution of cases (n = 50).

Age group (years)	Number of cases	Percentage (%)
0–10	1	2
11–20	9	18
21–30	16	32
31–40	10	20
41–50	6	12
51–60	4	8
61–70	3	6
>70	1	2

**Table 3:** Sex distribution of cases (n = 50).

Gender	Number of cases	Percentage (%)
Male	39	78
Female	11	22

Table 4 shows the categorization of cases of skin lesions based on the histopathological examination of skin biopsies into various categories. Out of 50 skin biopsies, 33 (66%) cases were of non-neoplastic skin lesions, 14 (28%) cases were of neoplastic skin lesions, and three (6%) cases were inconclusive.

**Table 4:** Type of skin lesions based on histopathology (n = 50).

Skin lesions	Number of cases	Percentage (%)
Non-neoplastic	33	66
Neoplastic	14	28
Inconclusive	3	6

**Figure 1:**
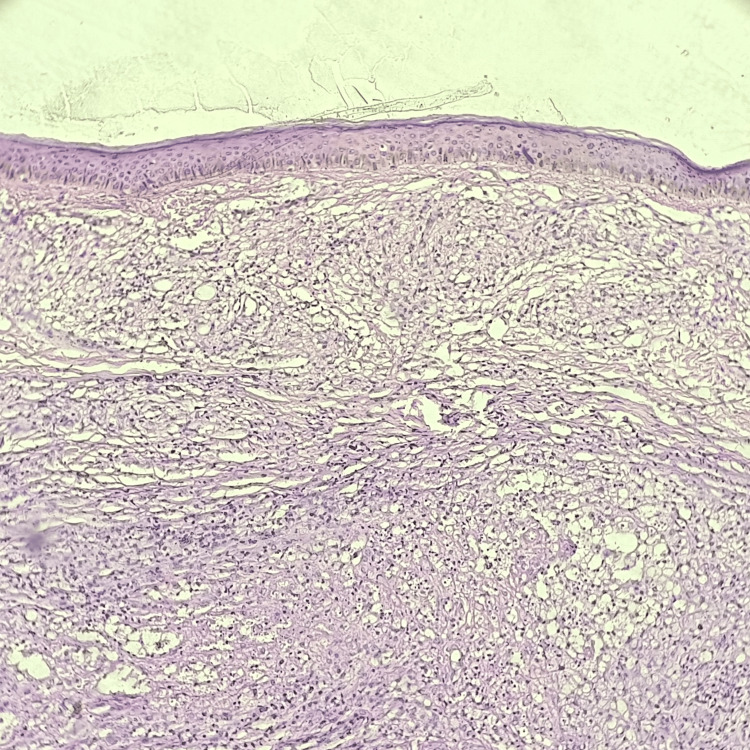
Hematoxylin and eosin-stained photomicrograph of lepromatous leprosy showing atrophic epidermis and dermis showing histiocytes.

**Figure 2:**
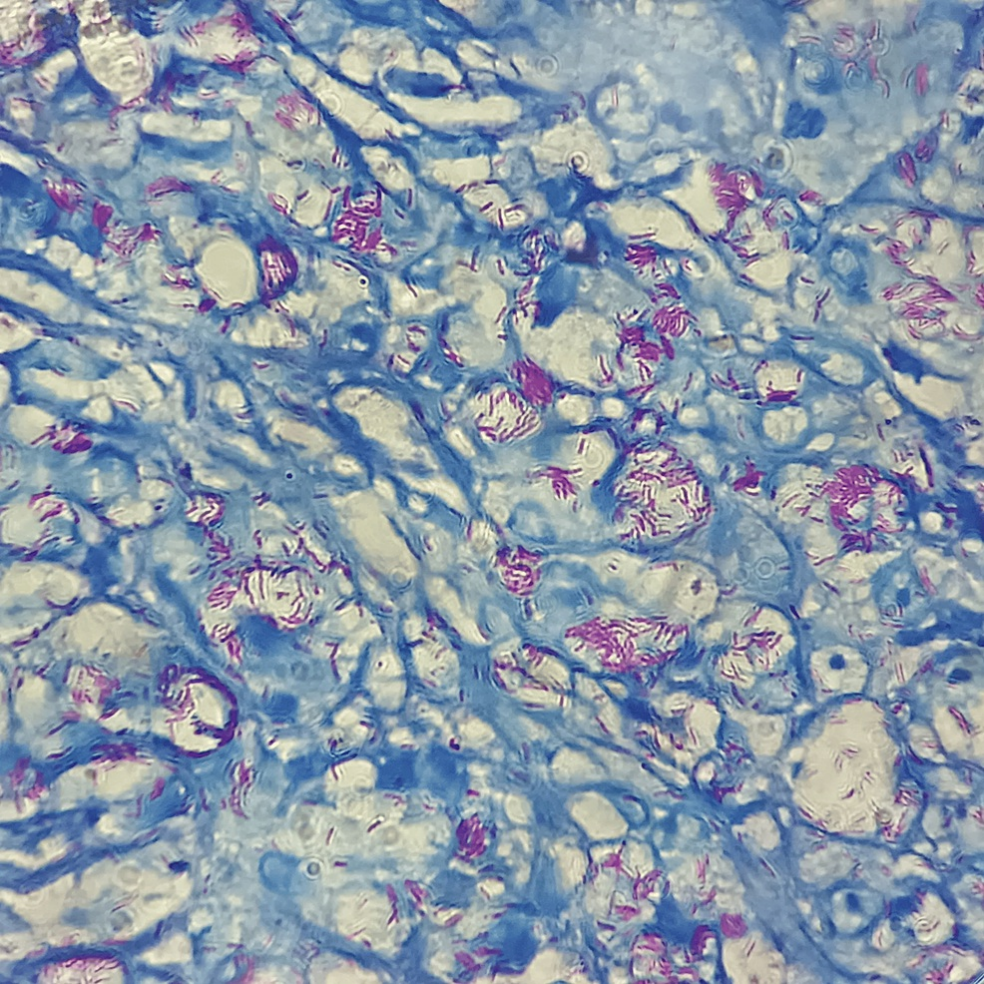
Fite-Faraco-stained photomicrograph of lepromatous leprosy showing numerous acid-fast bacilli.

**Figure 3:**
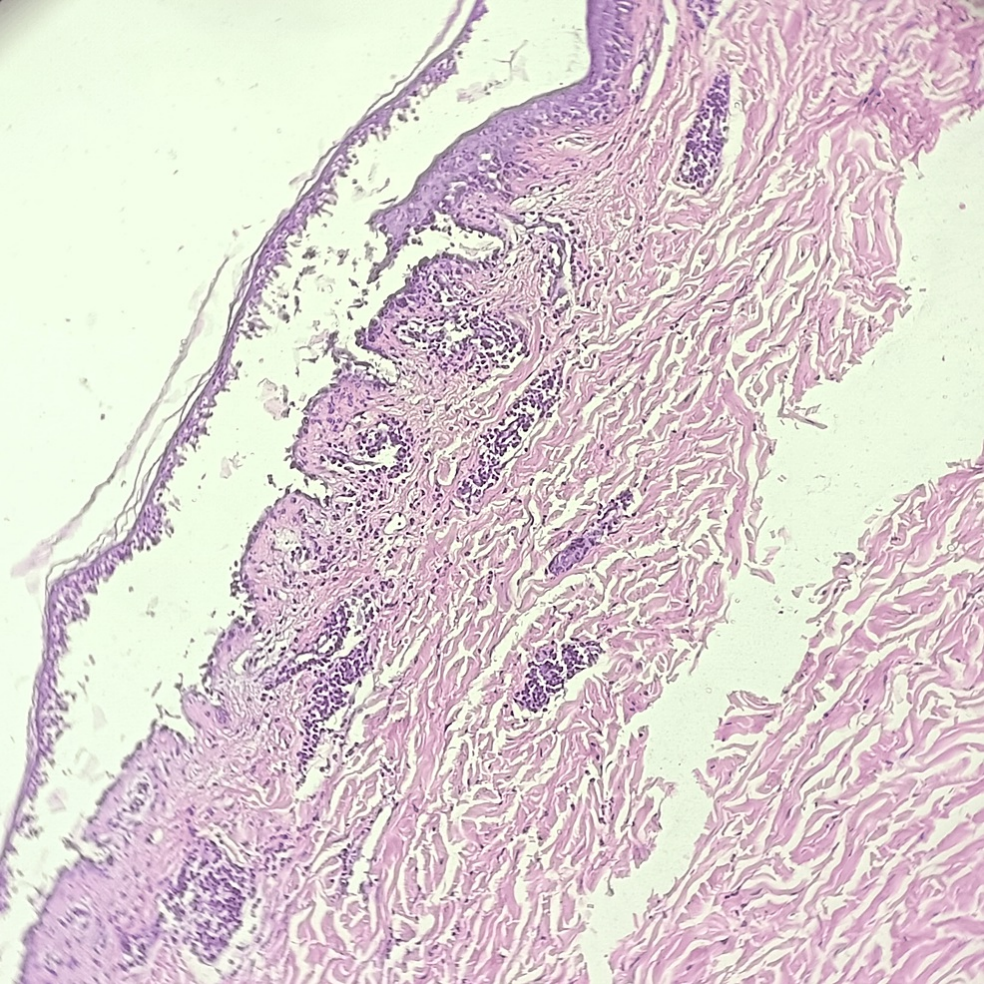
Hematoxylin and eosin-stained photomicrograph of pemphigus vulgaris showing intraepidermal blister in the suprabasal plane.

**Figure 4:**
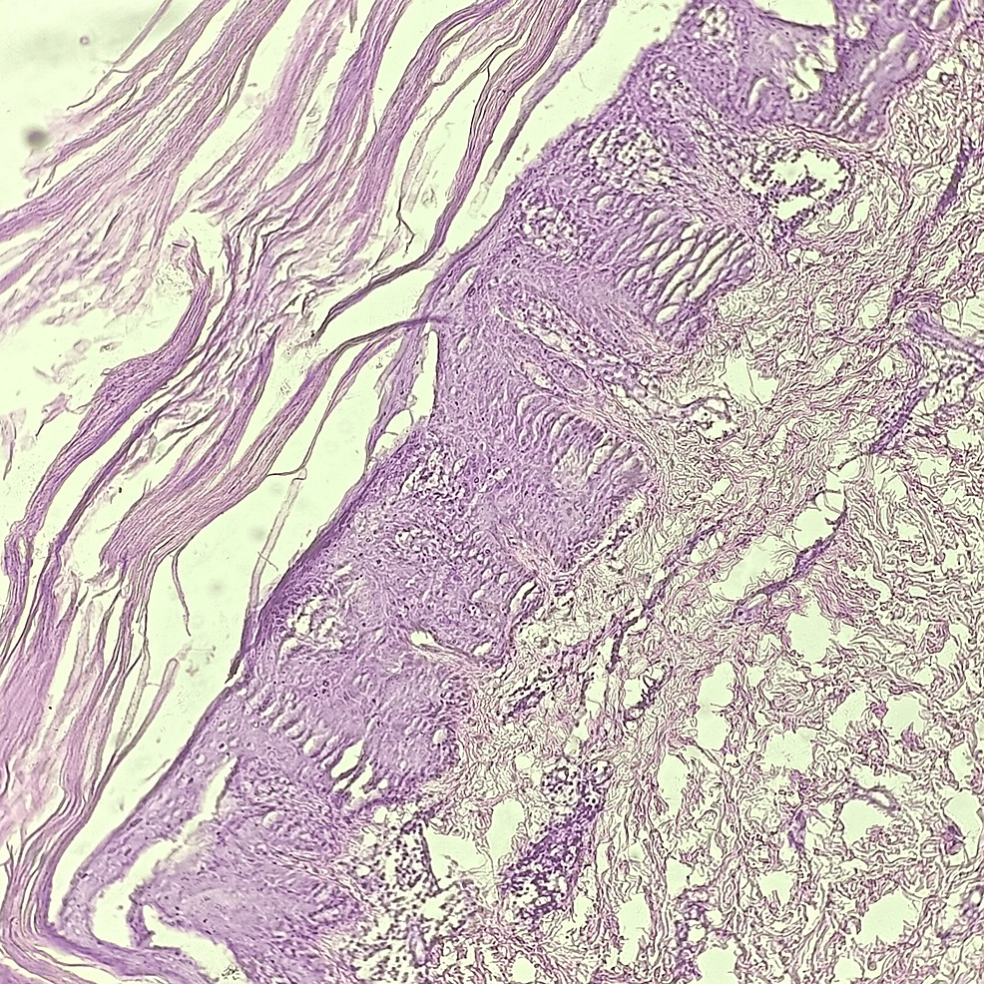
Hematoxylin and eosin-stained photomicrograph of psoriasis showing hyperkeratosis and parakeratosis.

Table 5 shows that microbial diseases were the most prevalent non-neoplastic type comprising 16 (32%) cases, followed by non-infectious vesiculobullous skin diseases and vesiculopustular skin diseases with eight (16%) cases, non-infectious papular and squamous skin lesions with five (10%) instances, and vascular disease with two (4%) cases. Connective tissue disease and inflammatory disease were the least frequently observed with one (2%) case. Among the microbial diseases, leprosy (Figures 1, 2) was the most prevalent disease, occurring in 12 (24%) cases, followed by tuberculosis in two (4%) cases. Sporotrichosis and chromoblastomycosis were the least frequent microbial diseases with one (2%) instance. Among the non-infectious vesiculobullous and vesiculopustular diseases, pemphigus vulgaris (Figure 3) made up the majority of vesiculobullous diseases, accounting for five (10%) cases, followed by subepidermal bullous disease, which was present in two (4%) cases. Psoriasis (Figure 4) was the most prevalent disease among non-infectious erythematous papular and squamous skin lesions, seen in two (4%) instances, followed by lichen planus, pityriasis rosea, and urticaria with one (2%) case each.

**Table 5:** Distribution of non-neoplastic skin lesions (n = 33).

Skin lesions	Number of cases	Percentage (%)
Non-infectious vesiculobullous and vesiculopustular diseases	8	16
Pemphigus vulgaris	5	10
Subepidermal bullous disease	2	4
Spongiotic dermatitis	1	2
Non-infectious erythematous papular and squamous lesions	5	10
Psoriasis	2	4
Lichen planus	1	2
Pityriasis rosea	1	2
Urticaria	1	2
Microbial diseases	16	32
Leprosy	12	24
Tuberculosis	2	4
Sporotrichosis	1	2
Chromoblastomycosis	1	2
Connective tissue disease	1	2
Discoid lupus erythematous	1	2
Vascular disease	2	4
Leucocytoclastic vasculitis	2	4
Inflammatory disease	1	2
Atopic dermatitis	1	2

**Figure 5:**
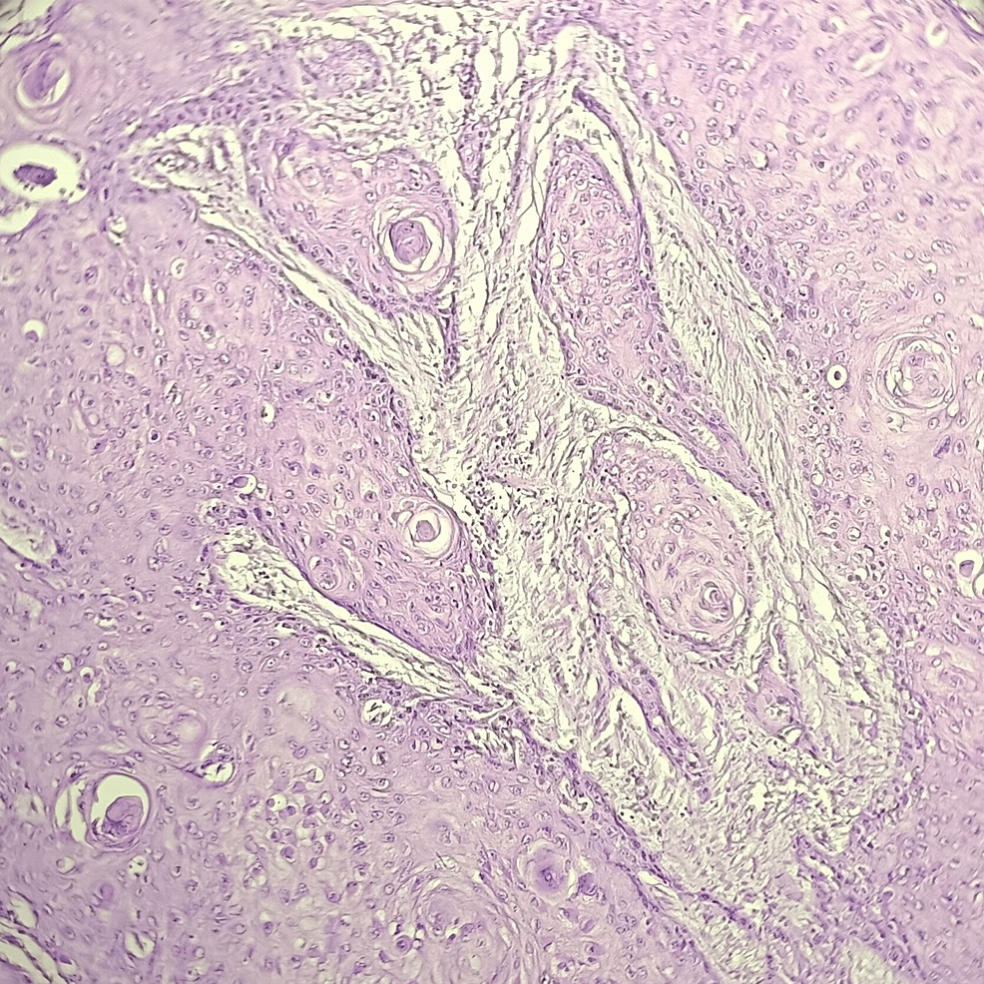
Hematoxylin and eosin-stained photomicrograph of squamous cell carcinoma showing marked keratinization.

**Figure 6:**
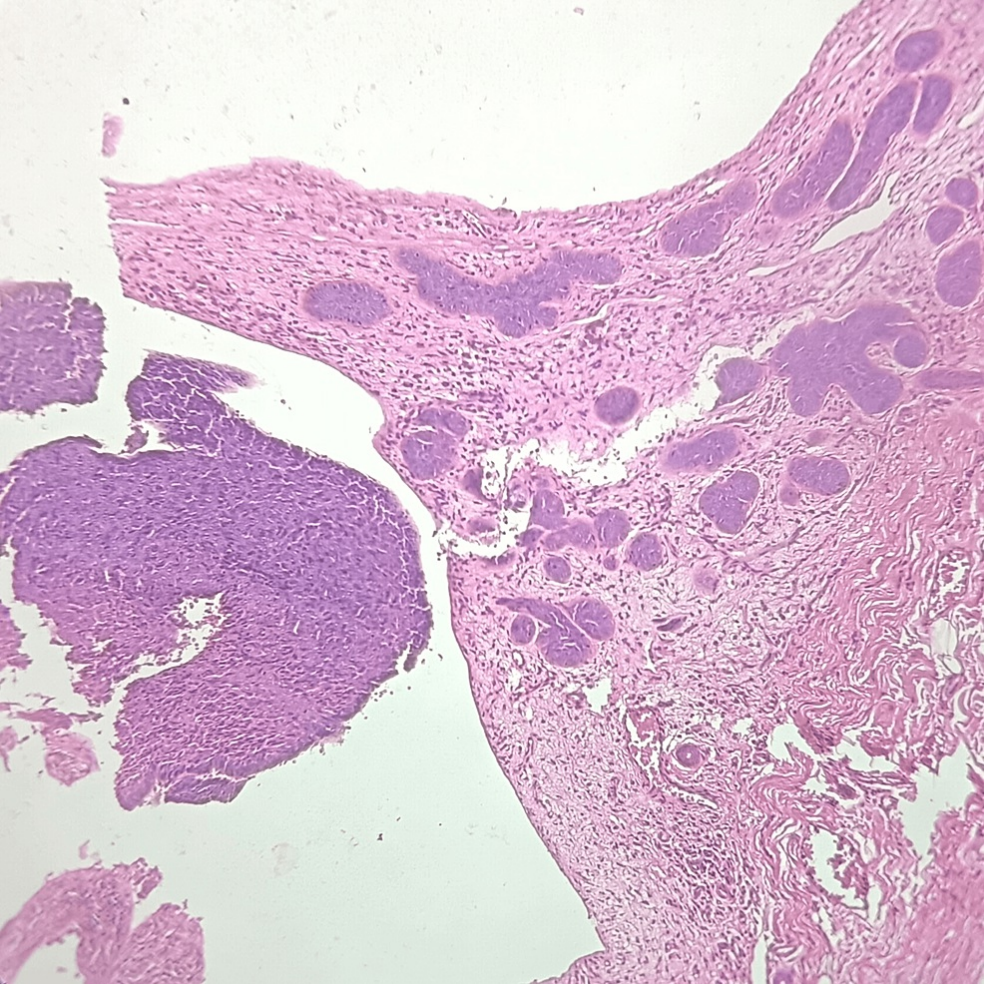
Hematoxylin and eosin-stained photomicrograph of basal cell carcinoma showing nest of basaloid cells.

Table 6 shows that squamous cell carcinoma (Figure 5) was the most frequent neoplastic lesion of the skin seen in seven (14%) cases, followed by basal cell carcinoma (Figure 6) in five (10%) cases. Whereas melanoma was the least frequently found neoplastic skin lesion with two (4%) cases.

**Table 6:** Distribution of non-neoplastic skin lesions (n = 14).

Skin lesions	Number of cases	Percentage (%)
Squamous cell carcinoma	7	14
Basal cell carcinoma	5	10
Melanoma	2	4

## Discussion

Skin lesions have a wide range of clinical and histological characteristics. The best method for identifying skin lesions is histopathological examination. To make a final diagnosis, however, a thorough history with clinical examination findings, a close histopathological examination of a biopsy sample of the skin, and the association between clinical diagnosis and histopathological findings are necessary. A straightforward outpatient technique called a skin biopsy aids in the verification of the therapeutic diagnosis [7].

In our study, the age group of 21 to 30 years showed the highest occurrence of skin illness which is similar to the studies by Bezbaruah et al. [8] and Abubakar et al. [9], who identified the greatest incidence of skin diseases in people between the ages of 21 and 30. Adhikari et al. [10] discovered the greatest incidence of skin illness in the age group of 31 to 40 years. The youngest patient encountered in the present study was a four-year-old and the oldest case was an 80-year-old. The age range of the patients in this study was between the first and eighth decades, which is similar to the findings of Ayesha et al. [11], Mehar et al. [12], Yalla et al. [13], George et al, [14], Deepthi et al. [15], Gaikwad et al. [16], Bharadwaj et al. [17], Sushma et al. [18], and Gupta et al. [19]. Patients in studies by Mamatha et al. [20], Singh et al. [21], Agrawal et al. [22], Narang et al. [23], and Kafle et al. [24] ranged in age from the first to the seventh decade, while patients in studies by Gulia et al. [25] ranged in age from the first to the ninth decade.

In our study, of the 50 cases, 39 (78%) cases involved men and 11 (22%) cases involved women. This finding is consistent with the findings of a study conducted by Singh et al. [26], which found that 54.5% of the cases were male and 45.5% of the cases were women. Mehar et al. [12] likewise found that 56% of cases were male and 44% of cases were female. Male preponderance according to the pattern of sex distribution, which is consistent with research by Veldhurthy et al. [27], Dayal et al. [28], and Kumar et al. [29] in contrast to female predominance in Bezbaruah et al. [8] and Adhikari et al. [10].

In our analysis, non-neoplastic skin lesions made up 33 (66%) cases, a significant increase from the 14 (28%) cases of neoplastic lesions. Neoplastic lesions were seen as a more common type of skin injury than non-neoplastic lesions in Bezbaruah et al. [8] and Abubakar et al. [9]. The most prevalent non-infectious vesiculobullous and vesiculopustular vesiculopustular condition in our study was pemphigus vulgaris five (10%) cases. Adhikari et al. frequently reported spongiotic dermatitis as a lesion, which is in contradiction to our study’s findings. In our analysis, psoriasis was the most prevalent non-infectious erythematous papulosquamous lesion which is similar to similar to Agrawal et al. [22]. In contrast to our analysis, Reddy et al. [30] discovered lichen planus as the most prevalent non-infectious erythematous papulosquamous lesion.

Leprosy was a frequently seen infectious skin lesion in our study, which was similar to the findings of Agrawal et al. [22]. It shows that in the studied location, contagious skin diseases such as leprosy are more prevalent. The community should, therefore, be informed on how to prevent the spread of skin diseases similar to leprosy. Dermatophytosis was the most frequent infectious skin lesion in the study by Karn et al. [31] and Walker et al. [32] in Nepal, in contrast to our data. This observed variation might be the result of hot, humid weather in a specific region, which may be the reason for an increase in the prevalence of dermatophytosis, a fungal infection.

In our study, the most common histopathological diagnosis was found to be squamous cell carcinoma, accounting for seven (14%) cases; this is congruent with the findings of Ayesha et al. [11] and Achalkar et al. [33]. Basal cell carcinoma was identified by Agarwal et al. [22] and epidermal cyst by Bharadwaj et al. [17] as the most prevalent histopathological diagnosis among neoplastic lesions. In the present study, about three (6%) of the cases yielded a result that was not conclusive. Adhikari et al. [10] and Barman et al. [34] found that it was 4.5% in contrast.

Limitations

The lack of follow-up and the lack of clinicohistological correlation were the limitations of the current study.

## Conclusions

In our study, leprosy and squamous cell carcinoma were the most often seen non-neoplastic and neoplastic skin lesions, respectively. To decrease the occurrence of both of these diseases, community members should be taught how to avoid droplet transmission and repetitive exposure to ultraviolet light from the sun without protection. From dermatitis to malignant neoplasms, we saw a wide range of skin disorders. The significance of particular histomorphological characteristics lies in differentiating distinct skin lesions and is crucial in formulating the ultimate diagnosis of all these varied skin lesions. This emphasizes the importance of a histopathological analysis for appropriate patient care.
